# Efficacy and safety evaluation of acupoint embedding for patients with ulcerative colitis: A protocol of systematic review and meta-analysis

**DOI:** 10.1097/MD.0000000000021812

**Published:** 2020-08-21

**Authors:** Jinhua Lu, Jun Zhou, Lu Wang, Chun Zhong, Xu Chen, Bo Jia

**Affiliations:** a School of Basic medical science; b School of Acupuncture–Moxibustion and Tuina, Chengdu University of Traditional Chinese Medicine, Chengdu, China.

**Keywords:** acupoint embedding, ulcerative colitis, meta-analysis, systematic review, protocol

## Abstract

**Background:**

Ulcerative colitis is a recurring digestive disease characterized by inflammation in the intestinal tract, which seriously affects the life of the patient. In recent years, it has played a role in obesity, osteoporosis, and gastrointestinal disorders, and has received more and more attention. However, there are no clear conclusions about its effectiveness and safety in the treatment of UC.

**Method and analysis:**

This systematic review will collect 7 databases, including Web of science, Pubmed, Embase, VIP, Wanfang, CNKI, and the Chinese Biomedical Literature Database (CBM), to collect all eligible RCTs from database inception to December 31, 2019. The 2 researchers will rigorously follow the selection process, including study screening, data extraction, and quality assessment. The primary outcome is clinical effectiveness. The main software used in this study is Review Manager V5.3 software.

**Result:**

This study will provide a meaningful and comprehensive evaluation on the effectiveness and safety of acupoints embedding for UC.

**Conclusion:**

This meta-analysis was designed to provide clinicians with valid evidence regarding acupoint embedding for UC.

**INPLASY registration number:**

INPLASY202040166.

## 1 Introduction

Ulcerative colitis (UC) is one of the main types of inflammatory bowel disease, associated with abdominal pain, diarrhea, and bloody stools. In addition, joints, eyes, skin, or liver may also be infected.^[1,2]^ UC occurs on the surface of the intestinal mucosa, starting the rectum and then spreading to the entire colon.^[3,4]^ In recent years, it is becoming a global disease, publications showed that UC is not only occurring in Western countries,^[5–7]^ but is also increasing in developing regions such as Asia and Africa, 1 study showed that there was a six-fold increase of incidence from 1986–1988 to 2004–2006 in Hong Kong.^[8]^ About the pathogenesis of UC, there is still no specific clinical conclusion, but most people think it is related to environment, genetics and immune.^[4,9,10]^ In addition, recent studies focused on gastrointestinal barrier function and gut microbes which may play an important role in causing the disease.^[11–13]^

Now, medicine and surgery are the common therapies for UC.^[4,14,15]^ However, recurring condition of UC and adverse effects are still problems to clinicians. ^[4,6,16]^ Some studies have showed that there is nausea, vomiting, headache, and anemia during oral medications.^[14,15]^ Thus, safe and effective therapies are needed to be discovered. Acupuncture, a traditional Chinese treatment, demonstrates its superiority in UC. It increases beneficial flora in the body, such as Lactobacillus and Bifidobacterium, while inhibiting harmful flora, such as Bacteroidetes and Clostridium perfringens.^[17]^ In addition, animal and human experiments also had shown that stimulation of skin muscles can affect gastrointestinal function.^[13,17,18]^ Acupoints catgut embedding evolved from acupuncture, it inherited the advantages of acupuncture which are definitive efficacy and few negative. Remarkably, it takes less time to heal but works for longer.^[19,20]^ So we will organize, analyze, summarize studies that we could find on all databases about acupoints catgut embedding for UC to provide a clear and significant evidence for clinicians.

## 2 Method

This protocol had been registered in International Platform of Registered Systematic Review and Meta-analysis Protocols (INPLASY), the registration number is INPLASY202040166, and the DOI number is 10.37766/inplasy2020.4.0166.

### 2.1 Inclusion criteria

#### 2.1.1 Type of studies

We will only include randomized controlled trials (RCTs) of acupoints catgut embedding for UC, regardless of reviews, protocols, animal experiments, case studies, non-therapeutic clinical studies.

#### 2.1.2 Types of participants

All participants were clinically diagnosed with UC, without restrictions on the TCM classification of UC, such as age, sex, disease duration, and race. But some special patients will not be included even if they meet the clinical criteria for UC, such as pregnant or nursing women, people with severe heart, liver or lung disease, those with the history of major trauma surgery, and local skin injuries at specific acupoints.

#### 2.1.3 Type of intervention

##### 2.1.3.1 Acupoints catgut embedding intervention

The observation group only include acupoints catgut embedding, and we will no restrictions on the equipment, frequency, course of the treatment, depth of needling, numbers of acupuncture points.

##### 2.1.3.2 Comparison intervention

The control group chose to take Western medicine, enema, moxibustion, placebo, Chinese herbal medicine, or other treatments regularly.

### 2.2 Type of outcomes

#### 2.2.1 Primary outcomes

The primary outcome is clinical effectiveness. It is based on the consensus opinion of diagnosis and therapy of UC proposed by the inflammatory bowel disease (IBD) group of the gastroenterology branch of Chinese medical association in 2018, and there is 3 levels: relief, effective, ineffective.^[21]^ The total effectiveness rate is a percentage, which is the ratio of the sum of the number of mitigators and effective people to the total number.

#### 2.2.2 Secondary outcomes

The secondary outcomes include inflammatory cytokines, the Baron and Mayo scores of UC.^[22,23]^ Inflammatory cytokines include IL-6, TNF-α, etc. The Baron score uses 0-4 to show the severity of the mucosa under colonoscopy. The Mayo score uses 0-3 to show a change of symptoms, including: stool frequency, rectum bleeding, findings of flexible proctosigmoidoscopy, and physicians global assessment. In addition, adverse reaction rate is an important indicator of safety.

### 2.3 Search Strategy

The computer will index RCTs trials on acupoints catgut embedding for UC published from build to December 31, 2019 and restrict the language to English and Chinese. We will search 7 databases, including 4 Chinese databases: VIP, Wanfang, CNKI, and the Chinese Biomedical Literature Database (CBM), and 3 English databases: the Web of Science, Pubmed, and Embase databases. Depending on the inclusion criteria, the following search words will be used: acupoint catgut embedding, acupoint therapy, ulcerative colitis, clinical trial, randomized controlled trial, and so on. (Table 1 shows the search strategy in Pubmed). Other databases will be searched in the same way as this one, or slightly modified to fit different databases.

**Table 1:**
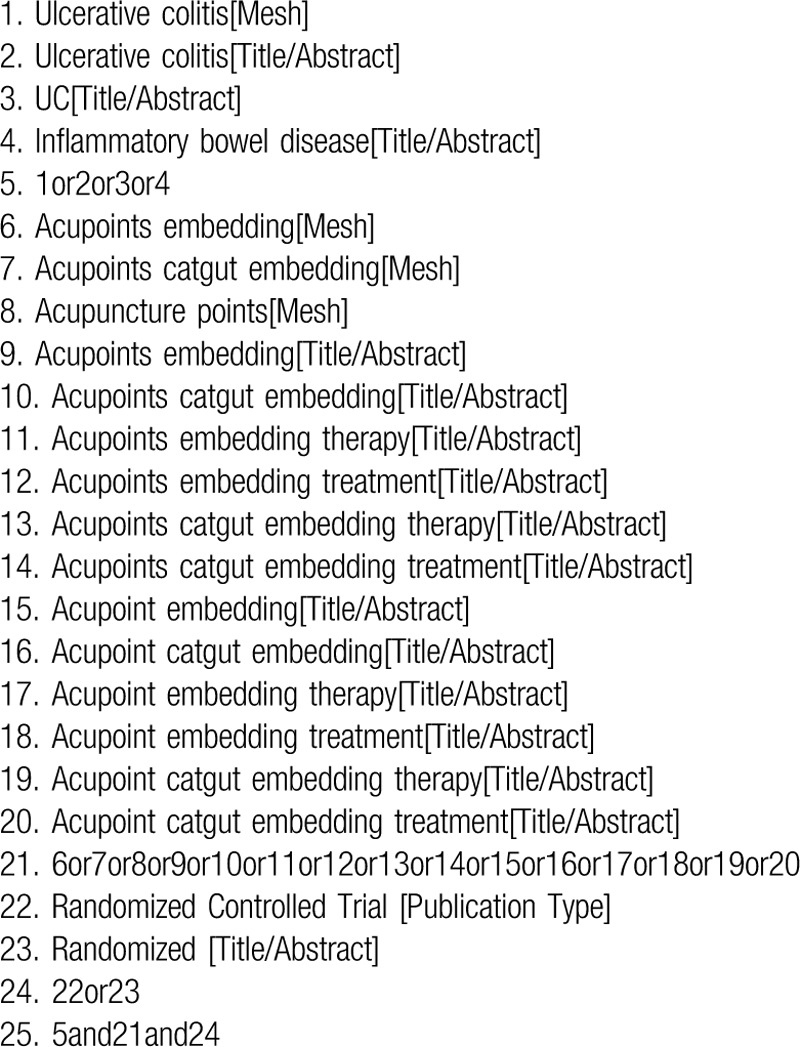
The search strategy in Pubmed.

### 2.4 Studies selection and data extraction

**Figure 1:**
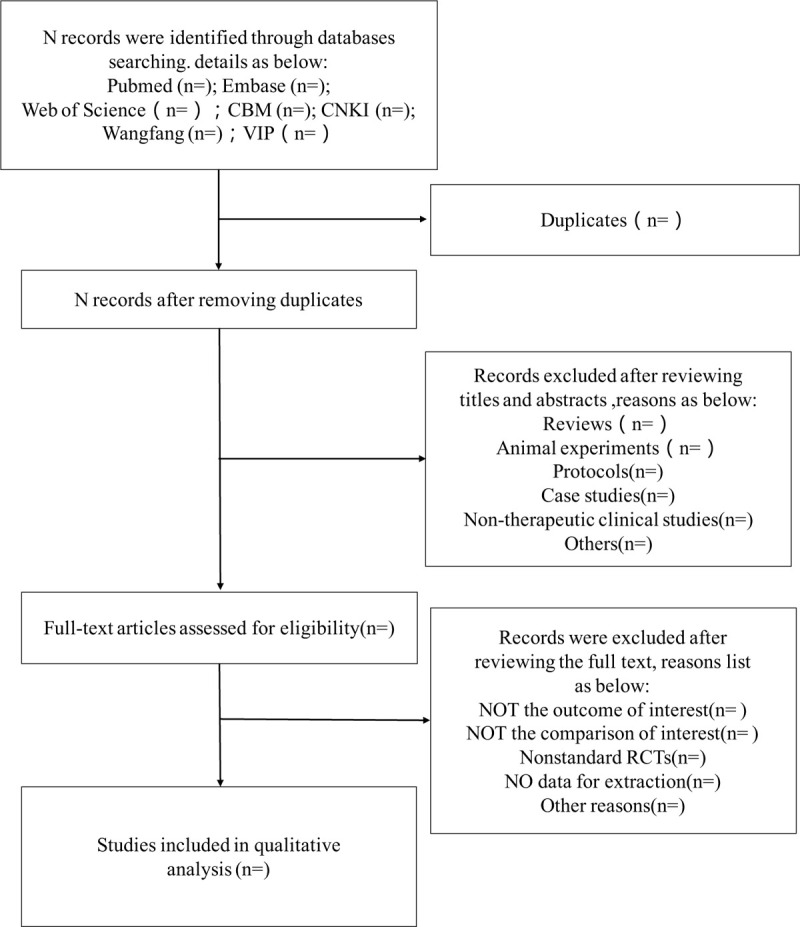
Flow diagram of the selection process.

Firstly, all studies should be imported into Endnote in order to remove duplicates, and then 2 researchers will meticulously skim the title and abstract to include studies that meet the inclusion criteria. After this, 2 investigators will carefully read the full text of the included studies and then use a uniform data extraction table to extract important information. The table includes time, authors, interventions, outcomes, adverse effects, etc. At all steps, the 2 have to work independently and if there a disagreement between 2 people, it will be decided by a group. This selection process will follow the PRISMA guidelines as shown in Figure 1.

### 2.5 Assessment of risk of bias

Two researchers will evaluate the quality of RCTs by using the risk assessment tool recommended in Cochrane Handbook 5.3. This evaluation includes 6 factors: generation of random sequences, blinding of investigators and participants, blinding of study results, completeness of outcome data, selectivity in reporting of results, and other biases. If there are missing or unclear data, we will attempt to contact the original authors by email. If no reply is received or the authors have not saved the original data, we will analyze only the data that are useful in the literature or analyze the missing data in the discussion.

### 2.6 Data analysis

Revman 5.3 software will be used to combine and analyze the results of all the studies. This study involves bicategorical and continuous variables. The relative risk (RR) is used as an effect measure in the bicategorical variables and the mean differences (MD) in the continuous variables, and the software is able to obtain the point estimates and the 95% confidence interval (CI) for the 2. *I*^2^ is an important index for making the heterogeneity judgment. If *I*^2^ < 50%, a fixed effects model is used; if *I*^2^ ≧ 50%, a random effects model is used. For each combined analysis, the test of heterogeneity is measured using the cardinality statistic. If *I*^2^ ≧ 50%, substantial heterogeneity is considered to be present. If heterogeneity is present, we will analyze the cause through subgroup analysis and sensitivity analysis.

### 2.7 Grading the quality of evidence

We will evaluate the risk based on 4 areas: bias, inconsistency, indirectness, inaccuracy, and publication bias, and then grade the evaluate of results: high, moderate, low, and very low.

### 2.8 Ethics

This is a systematic review and will not involve patients personal data, so ethical consent is not required.

## 3 Discussion

In recent years, more and more researches have been devoted to UC, but there still is not a golden standard for treatment. Ulcerative colitis (UC) which characterize by recurring inflammation of the bowel not only brings financial strain, but also affects patients quality of life (QoL).^[1,5,24]^ The theory of acupoints embedding is from Chinese medicine, which can balance the bodys yin and yang by continuously stimulating acupuncture points. It is getting more and more attention as a form of therapy. And acupoints embedding plays a role in osteoporosis, insomnia, obesity.^[19,25,26]^ Acupoints embedding has been a routine treatment in Chinese, but there is still no clear evidence of its effectiveness and safety in UC. Therefore, it is significant to study these of acupoints embedding for UC. We will refine this protocol to get useful results and provide Chinese solutions to medical professionals around the world.

## Author contributions

**Conceptualization:** Jinhua Lu, Lu Wang, Jun Zhou.

**Data curation:** Jinhua Lu.

**Funding acquisition:** Chun Zhong.

**Investigation:** Lu Wang, Bo Jia.

**Methodology:** Xu Chen, Jun Zhou.

**Project administration:** Jinhua Lu, Xu Chen.

**Software:** Jun Zhou.

**Supervision:** Chun Zhong.

**Validation:** Lu Wang.

**Writing – original draft:** Jinhua Lu, Lu Wang.

**Writing – review & editing:** Jun Zhou, Bo Jia.
